# Automated Test Assembly for Multistage Testing With Cognitive Diagnosis

**DOI:** 10.3389/fpsyg.2021.509844

**Published:** 2021-05-06

**Authors:** Guiyu Li, Yan Cai, Xuliang Gao, Daxun Wang, Dongbo Tu

**Affiliations:** ^1^School of Psychology, Jiangxi Normal University, Nanchang, China; ^2^Department of Curriculum and Instruction, East China Normal University, Shanghai, China; ^3^School of Psychology, Guizhou Normal University, Guiyang, China

**Keywords:** cognitive diagnosis, computer multistage test, automated test assembly, cognitive diagnosis modules, heuristic algorithms

## Abstract

Computer multistage adaptive test (MST) combines the advantages of paper and pencil-based test (P&P) and computer-adaptive test (CAT). As CAT, MST is adaptive based on modules; as P&P, MST can meet the need of test developers to manage test forms and keep test forms parallel. Cognitive diagnosis (CD) can accurately measure students’ knowledge states (KSs) and provide diagnostic information, which is conducive to student’s self-learning and teacher’s targeted teaching. Although MST and CD have a lot of advantages, many factors prevent MST from applying to CD. In this study, we first attempt to employ automated test assembly (ATA) to achieve the objectives of MST in the application of CD (called CD-MST) via heuristic algorithms. The mean correct response probability of all KSs for each item is used to describe the item difficulty of CD. The attribute reliability in CD is defined as the test quantitative target. A simulation study with the G-DINA model (generalized deterministic input noisy “and” gate model) was carried out to investigate the proposed CD-MST, and the results showed that the assembled panels of CD-MST satisfied the statistical and the non-statistical constraints.

## Introduction

The computer multistage adaptive test (MST), as a “balanced compromise” between CAT and P&P, not only can provide high measurement accuracy as CAT (Kim et al., 2015) but also can meet the need of test developers to manage test forms and keep test forms parallel. CAT is an item-level adaptive test; however, MST sets a module to manage items and to be adaptive at the module level. MST allows subjects to modify the item answers in the current stage, which is beneficial to reduce the examinees’ test anxiety and improve the measurement accuracy. Compared with CAT, MST has many inherent advantages: (1) CAT does not allow examinees to modify item answers, which leads to the lack of test control and generates test anxiety for the examinees. MST can allow examinees to modify their item answers in the current stage, which helps alleviate test anxiety while avoiding measurement mistakes caused by errors. (2) CAT pursues the items with the maximum information during an adaptive stage, which will result from overexposure of items with high information. In contrast, MST can effectively enhance the use rate of item bank and control item exposure rate by constructing several parallel panels. (3) CAT is not good at balancing the non-statistical characteristics of the test [e.g., content constraints, item types, enemy item (there are clues to the answers between the items), word count, etc.]. MST can manage both statistical and non-statistical characteristics, which can greatly improve content validity and measurement precision. (4) Compared with CAT online testing, MST preassembles a test before performing the test administration, which can help test developers better manage a test. Because of these benefits, many high-stake tests have switched from the CAT mode to the MST mode (Wang et al., 2015), such as the United States National Education Progress Assessment (NAEP), the US Graduate Entrance Examination (GRE), the Program for the International Assessment of Adult Competencies (PIAAC), and other large examinations (Yamamoto et al., 2018).

Currently, the classical test theory (CTT) and the item response theory (IRT) have been widely used in education, psychology, psychiatry, etc. However, both the CTT and the IRT mainly focus on the examinees’ trait or competency level, and therefore, they cannot provide further information on the internal psychological processing, processing skills, and cognitive structures hidden behind the results of the test scores (Embretson and Yang, 2013). Unlike the CTT and IRT, which can only provide an examinee’s score, cognitive diagnosis (CD) can further report the examinee’s knowledge states (KSs), cognitive structures, and other diagnostic information. This feature of CD can help teachers carry out targeted teaching and promote education development. Currently, CD, as a representation of the new generation testing theory, has widely attracted the attention of researchers and practitioners and has become an important area of psychometrics research.

Recently, researchers consider that the cognitive diagnostic model can be applied to the MST (von Davier and Cheng, 2014). It is called CD-MST, a new test mode that combines the advantages of CD and MST. First, it can present items with the function of CD and help test developers to manage a CD test before administering it. Second, CD-MST can provide rich diagnostic information to each examinee and guide students and teachers to self-study, adaptive study, individual teaching, remediation teaching, etc. Third, CD-MST is adaptive in modules, where examinees can review and revise item answers. That is closer to the examination scene and helps to reduce examinees’ test anxiety. Finally, the adaptive CD-MST can use fewer items to provide immediate and accurate cognitive diagnostic feedback information, and the advantages of CD-MST are especially highlighted in classroom assessment or practice.

Although CD-MST has many advantages, some problems make its assembly infeasible: (1) Item difficulty index. In MST with the IRT, the item difficulty parameter *b* can accurately indicate the examinees’ traits value θ because they are in the same scale. At this point, MST can use the *b* parameter to divide the item bank and assemble modules based on item difficulty. However, there is no item difficulty parameter in CD, and item parameters and examinee parameters are not set on the same scale. Even if the reduced reparameterized unified model (R-RUM; Hartze, 2002) has a completeness parameter based on the attribute, it is difficult to describe the item difficulty and to explain the relationship between the attribute master pattern and the item difficulty. Therefore, the key for CD-MST is to develop a new item difficulty index in CD. (2) Information or measurement precision index. MST with the IRT focus on a continuous variable. Fisher information, a typical statistic curving continuous variable, is used to ensure measurement precision and to control measurement errors, but CD measures discrete multidimensional variables, Fisher information is not suitable. In order to ensure the test reliability, accuracy, or to control measurement errors, selecting another robust statistical information index of CD is worth further study.

This study aimed to address this aforementioned issue and to develop a CD-MST framework. The rest of the paper is organized as follows. The MST framework is briefly introduced first. Then, the CD-MST framework is proposed, where two indexes, namely, the item difficulty index and the information (or measurement precision) index based on CD, and the automated test assembly (ATA) method for CD-MST are also proposed. Furthermore, the simulation study and the results were carried out to verify the proposed CD-MST framework. Finally, we discuss the limitations of this study and the further directions of CD-MST.

## MST Framework

### Multistage Adaptive Test

**FIGURE 1:**
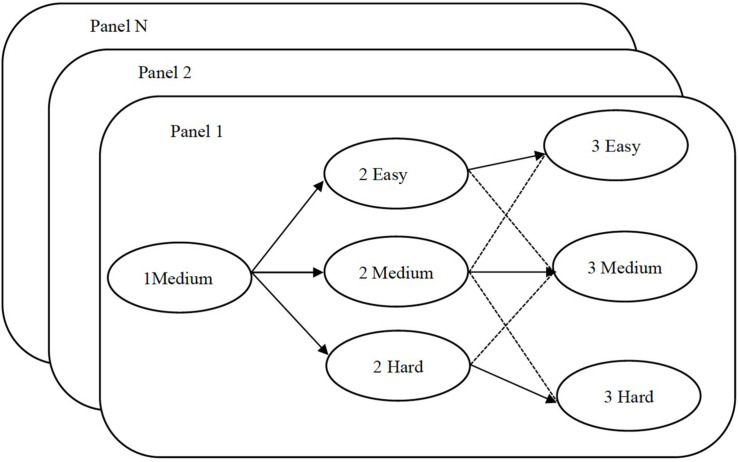
Three-stage multistage adaptive test (MST) of multiple parallel panels.

MST is built on several parallel panels. A complete panel includes the following elements: module, stage, and pathway, as shown in Figure 1. In MST, the test has three adaptive stages, and each stage contains several modules. Modules are composed by items that are according to certain test specifications and of different levels of item difficulty. In Figure 1, 1Medium indicates that the item difficulty of the first stage is moderate; 2Easy, 2Medium, and 2Hard indicate that the item difficulty of the second stage is easy, moderate, and difficult, respectively; and 3Easy, 3Medium, and 3Hard are analogous for the third stage. Panels 1, 2, and N represent the parallel test panels. When the test starts, examinees are randomly assigned to a pre-assembled test panel, and then according to their responses in the first stage, examinees are adapted to the module in the next stage that matches their ability. A series of modules responded by examinees is used to construct a response pathway. Each panel has seven test pathways, as shown in Figure 1 (see the arrow’s direction in Figure 1). Among them, the solid line arrows (e.g., 1Medium + 2Easy + 3Easy, 1Medium + 2Medium + 3Medium, and 1Medium + 2Hard + 3Hard) denote the three primary pathways that examinees are most likely to adapt, whereas the dotted lines denote the four secondary pathways (Luecht et al., 2006).

Parallel test panels are the core of MST. It needs to meet the requirements of the test specifications. Test specifications include both the statistical targets (e.g., test information) and the non-statistical targets (e.g., content constraint), which ensure that each test panel has precise reliability and validity. In MST, the statistical and non-statistical targets mainly relate to the target test information function (TTIF), the test length, the number of stages and modules, the content balance, the exposure control, etc. However, these factors are not independent from each other when building panels, but rather are tightly integrated into the MST architecture (Yan et al., 2014). Like in linear tests, to ensure the safety of the test and the use rate of the item bank, MST researchers hope to set up multiple parallel panels (Samejima, 1977). In linear tests, an item is preliminarily formed into a fixed test form. When test information and other measurement targets are sufficiently similar, it can be assumed that these pre-assembled test forms are parallel. Test pathways in MST are the same as test form in the linear test. However, modules in MST have different difficulty levels; pathways constituted by modules are often not parallel in statistical information. Automated test assembly is a way to achieve parallelism between tests and to meet the test specifications. We build parallel panels according to specific test specifications. When two different pathways in two different panels are parallel, the panels can be viewed as parallel (Yan et al., 2014). It is important to note that when parallel pathways are set up for the test specification, it is not necessary to have parallelism between the modules (Yan et al., 2014).

MST assembly should meet the following three goals: (1) the module has a clear information curve enough to distinguish between the different stages of tests; (2) the information of corresponding pathways between panels is similar to ensure that the panel is parallel; and (3) each pathway of each panel satisfies non-statistical constraints (Yan et al., 2014).

### Multistage Adaptive Test Design

The MST design includes the number of panels, the number of stages in panels, the number of modules in stages, the number of items in modules, the level of item difficulty, etc. (Yan et al., 2014). It also involves the assembly strategies and the assembly methods. The assembly strategies determine the item difficulty levels, the content balance, and other elements parallel in modules or pathways. The ATA method ensures that these elements (statistical and non-statistical constraints) are parallel on panels. Statistical constraints are initially determined by the item difficulty and discrimination of the CTT (Gulliksen, 1950), and now, the test information function (TIF) has become the main form of statistical characteristics. The target TIF of IRT usually uses the Fisher information, which was described in detail by Luecht and Burgin (2003). Besides, the statistical constraints of the target TIF need to consider whether the item bank meets test specifications. For example, the quality and the number of items in an item bank are required to provide a great TIF.

### Multistage Adaptive Test Assembly Strategies

After the MST design is completed, the parallel panels need to be assembled by using MST assembly strategies, which involve a bottom–up strategy and a top–down strategy (Luecht and Nungester, 1998).

In the top–down strategy, parallel panels are based on the pathways. Several parallel panels are constructed from an item bank, and the corresponding pathways in different panels are parallel. Here, parallel includes the statistical constraints (target TIF) and the non-statistical constraints. The parallel pathways contain two types of pathways, namely, the three primary parallel pathways (see the three thick line pathways in Figure 1) and all the parallel pathways. When the three primary parallel pathways are used, the test specification is divided into three primary pathways, and other pathways randomly assemble with the item difficulty. Because the three primary pathways represent the majority answer pathways of examinees, the panels only need to ensure that the three primary pathways are parallel in different panels. When all parallel pathways are used, test specifications are divided into all possible pathways. When building parallel MST panels with a top–down strategy, we set the target TIF for the entire test and assign the non-statistical constraint to the pathways.

In the bottom–up strategy, parallel panels are based on the modules. The assembly of parallel modules is parallel between the statistical constraints (target TIF) and the non-statistical constraints. When the modules are parallel, we can mix the parallel modules to assemble multiple parallel panels. As the modules are parallel to each other, the corresponding pathways of panels will automatically be parallel. When using the bottom–up strategy to set up parallel MST panels, we set different target TIF to modules with different item difficulties. In contrast, non-statistical constraints are allocated to each module (Yan et al., 2014).

## The CD-MST Framework

### Cognitive Diagnosis Combined With Multistage Adaptive Test

As mentioned above, CD-MST combines the advantages of both CD and MST. Similar to MST, CD-MST also includes similar elements or parts, such as the panel, module, stage, pathway, CD-MST design, assembly strategies, and assembly methods. The main difference between MST and CD-MST is that the latter can provide additional rich diagnostic information for each examinee. The information can provide insight on self-study, adaptive learning, and remediation teaching.

In the *Introduction* section, we noted some indexes in the test assembly for MST, such as the item difficulty and the Fisher information describing continuous variables and reflecting the measurement precision. They may not be suitable for CD-MST framework because CD mainly focuses on the multidimensional and discrete cognitive attributes or KSs. To develop a CD-MST framework, we propose a new assembly method for CD-MST as below.

### CD-MST Assembly Strategy

The ATA method is the main algorithm for MST, which currently contains heuristic methods, linear programming methods (Zheng et al., 2012), and Monte Carlo methods (Belov and Armstrong, 2005). The linear programming algorithm can successfully complete the test requirements and strictly meet all the test assembly constraints (e.g., content constraints and enemy items) (Zheng et al., 2012). However, solving the 0–1 linear programming problem is very complex (Theunissen, 1989) and time consuming. With the test constraint complexity increasing, the limited item bank cannot meet all the test constraints. It will induce infeasible problems about overconstrained optimization and lead to test assembly failure.

According to the heuristic algorithms, the test assembly is decomposed into a series of local optimization problems. Each local optimization problem is chosen as a single item for tests (Ackerman, 1989; Lord, 1977). It uses statistical information as a central function (such as the TIF) and considers non-statistical constraints. Heuristic algorithms are less computationally intensive and always effectively complete the test assembly (Zheng et al., 2012); therefore, we used heuristic algorithms to assemble a test for CD-MST in this study.

### Item Difficulty Index for Cognitive Diagnosis

In this study, the mean correct response probability of all KSs of one item was used to indicate the item’s difficulty. The attribute mastery pattern in an item is finite and known when the Q-matrix is fixed. Therefore, the mean correct response probability of all KSs can reflect this item’s difficulty levels, and it is expressed as:

(1) $$Diff_{j}=\frac{\sum_{c=1}^{2^{K}}P_{j}\left(\alpha_{c}\right)}{2^{K}},$$

where *Diff*_*j*_ is the difficulty parameter of item *j* on CD, K is the number of attributes, and *P*_*j*_(*a*_*c*_) is the correct response probability on item *j* for individuals with the KS of *a_c_*⋅*P*_*j*_(*a*_*c*_), which can be calculated by the item response function of CD models (such as the G-DINA model, see Equation 16). The lower the value of *Diff*_*j*_ is, the more difficult item *j* is.

To investigate whether this index can represent item difficulty, we compared *Diff*_*j*_ and the item difficulty parameter estimated by the IRT model (such as the Rasch model). We used the G-DINA model (for details, see Equation 16) to generate the response data (including 100 items, 1,000 individuals, and five independent attributes), and then we used the G-DINA model and the Rasch model to estimate the same response data, respectively. We calculated each item difficulty on CD via Equation 1 and the item difficulty parameter on Rasch model. The correlation coefficient of item difficulty between CD and IRT reached a value above 0.85 (*p* < 0.001), which clearly shows that the item difficulty based on CD had a significantly high correlation with the item difficulty on IRT. Therefore, the mean correct response probability of all KSs can be viewed as an item difficulty index under the CD framework.

### Reliability of Cognitive Diagnosis

Templin and Bradshaw (2013) proposed an empirical reliability index for CD. The reliability index defined the recalculation consistency using the tetrachoric correlation coefficient. They used the following steps to estimate the attribute reliability. (1) Calculate the marginal mastery probability of attribute *k* for examinee e ${\hat{p}}_{ek}$ by using CD models. (2) Establish the replication contingency table. For the binary attribute, four elements are calculated as follows:

(2) $$P\left({\alpha .}_{k1}=1;{\alpha .}_{k2}=1\right)=\frac{\sum_{e=1}^{N}{\hat{p}}_{ek}{\hat{p}}_{ek}}{N},$$

(3) $$P\left({\alpha .}_{k1}=1;{\alpha .}_{k2}=0\right)=\frac{\sum_{e=1}^{N}{\hat{p}}_{ek}\left(1-{\hat{p}}_{ek}\right)}{N},$$

(4) $$P\left({\alpha .}_{k1}=0;{\alpha .}_{k2}=1\right)=\frac{\sum_{e=1}^{N}\left(1-{\hat{p}}_{ek}\right){\hat{p}}_{ek}}{N},$$

(5) $$P\left({\alpha .}_{k1}=0;{\alpha .}_{k2}=0\right)=\frac{\sum_{e=1}^{N}\left(1-{\hat{p}}_{ek}\right)\left(1-{\hat{p}}_{ek}\right)}{N},$$

The attribute reliability was calculated by the tetrachoric correlation coefficient of α_.*k*1_ and α_.*k*2_, which also represents the re-test reliability of attribute *k*. More details can be found in Templin and Bradshaw (2013).

### Quantitative Targets for CD-MST

Quantitative targets include the test target reliability of CD, item difficulty, etc. In this study, the attribute reliability of the cognitive diagnostic model proposed by Templin and Bradshaw (2013) was used as a metric of the test reliability. This index provides attribute reliability to each cognitive attribute. In the study, the reason for using reliability to assemble the test is that a good reliability can reduce the measurement error and improve the reliability for the test. Reliability or information has always been used to measure the test reliability of both CTT and IRT. In CTT, the reliability coefficient was used to control test error. In IRT, information was used to control test error, but in CDM, the attribute mastery patterns are discrete variables. Based on the characteristics of CDM, Templin and Bradshaw (2013) proposed attribute reliability to control test error and ensure reliability. On the other hand, mainstream assembly algorithms in MST use test information function (TIF) to assemble test pathways, for example, Yang and Reckase (2020) used the Fisher information to assemble test for optimal item pool design in MST, and Xiong (2018) used the Fisher information in a hybrid strategy to construct MST. Yamamoto et al. (2019) used test characteristic curves (TCCs) in MST test design for PISA 2018. Whether these studies use reliability or information, the purpose is to control test errors and provide a greater reliability. Therefore, borrowing the ideas from the previous studies, we used attribute reliability to assemble tests and to control test errors because of the characteristics of CDM and MST.

## The Normalized Weighted Absolute Deviation Heuristic for CD-MST

The normalized weighted absolute deviation heuristic (NWADH; Luecht, 1998), a popular heuristic algorithm, has been applied to the MST assembly. The weighted deviations of constrained targets are used in this algorithm, and the deviation of each constraint is standard with the same scale (van der Linden and Glas, 2000). They also are compatible with multiple contents or classification dimensions, multiple quantitative targets, multiple test modules, and other complex test group issues, such as the enemy items (Luecht, 1998). Therefore, the NWADH is employed for the test assembly in CD-MST.

In NWADH, both statistical and non-statistical constraints are combined to set the objective function and to meet the current test requirement. With the selection of each item, the objective function is updated according to the measurement characteristics of the selected item, which is done until the test assembly is completed (Luecht, 1998). A well-designed test has a clear test specification so that measurement properties, quantitative targets, and other constraints should be considered in the test assembly. The statistical and non-statistical constraints for a test specification will be described in detail below.

Let *T*_*k*_ denote the target reliability of attribute *k* with test. $u_{k}^{j}$ denotes the observed reliability of attribute *k* in the test with a length of *J* items, which can be calculated by the tetrachoric correlation coefficient, and the difference of attribute reliability between the target attribute reliability and the observe attribute reliability can be calculated as follows:

(6) $$d^{J}=\sum_{k=1}^{K}\left|T_{k}-u_{k}^{J}\right|/K$$

In Equation 6, *J* denotes the selected items in the test, and *d^J^* represents the mean absolute deviation between the target attribute reliability and the observe attribute reliability with *J* items. When the new item was added to the test with *J* items, the test length is *J*+ 1 items. At this time, the difference of attribute reliability between the target attribute reliability and the observed attribute reliability can be calculated as Equation 7:

(7) $$d_{i}^{J+1}=\sum_{k=1}^{K}\left|T_{k}-u_{k}^{J+1}\right|/K;i\in R_{J}$$

In Equation 7, *R_J_* refers to the remaining items in the item bank after selecting *J* items. The item *i* is selected from *R_J_*. In order to meet statistical constraints, in CD-MST, the next item *i* of *R_J_* that makes $d_{i}^{j+1}$ with the smallest values was selected.

At the same time, in order to optimize the NWADH algorithm, we can transform the minimizing of the absolute deviation function in Equation 6 into the maximization, as follows:

(8) $$MAX\left(e_{i}\right)$$

where *e*_*i*_ is the “priority index” and is expressed as:

(9) $$e_{i}=1-\frac{d_{i}^{J+1}}{\sum_{i\in R_{J}}d_{i}^{J+1}};i\in R_{J}$$

In Equation 8, *e*_*i*_ denotes the priority index of item *i*. That means that CD-MST priority selects the items to make *e*_*i*_ with the maximum values in the remaining item bank *R_J_*.

Equations 6 and 9 are the NWADH algorithms (Luecht, 1998) when only considering the statistical quantitative target. However, a complete CD-MST also needs to consider non-statistical constraints such as content balance, item type, item answer, and other constraints. The NWADH algorithm can merge multiple content constraints (Luecht, 1998). When considering the content constraints, it is necessary to give a certain weight to constraints based on the test specifications. In general, the weight values depend on the test specifications that can be obtained by the pre-simulation (Luecht, 1998). The NWADH algorithm (Equation 9) contains the content constraints as follows:

(10) ei*=[1-diJ+1∑i∈RJdiJ+1]+ci∑i∈RJci;i∈RJ

where:

(11) $$c_{i}=v_{ig}W_{g}+\left(1+v_{ig}\right){\underline{W}}_{g},$$

(12) $${\underline{W}}_{g}=W^{\left[\max\right]}-\frac{1}{G}\sum_{i=1}^{G}W_{g}.$$

In Equation 10, *c*_*i*_ denotes the content constraint weight for each unselected item in the remaining item bank. In Equation 11, *g* denotes the total number of content constraints *g* = 1, …, *G*. *v*_*ig*_ = 0 indicates that item *i* does not contain the content constraint *g*, whereas *v*_*ig*_ = 1 indicates otherwise. *W*_*g*_ represents the weight of each content constraint *g*. ${\underline{W}}_{g}$ represents the mean weight of each content constraint *g*. In Equation 12, *W*^[*max*]^ represents the maximum weight values of *G* kinds of content constraints. In this study, the weight of the non-statistical constraints was according to the method proposed by Luecht (1998). The non-statistical constraints in the study were set as follows:

(13) $$if\sum_{i\in R_{j-1}}v_{i}\geq Z_{g}^{\left[\max\right]},thenW_{g}=1,$$

(14) $$if\sum_{i\in R_{j-1}}v_{i}<Z_{g}^{\left[\min\right]},thenW_{g}=2,$$

subject to the constraints,

(15) $$\sum_{i=1}^{I}v_{ig},g=1,\ldots ,G.$$

Let $Z_{g}^{\left[max\right]}$ represent the maximum constraint values of constraint *g*. $Z_{g}^{\left[min\right]}$ represents the minimum constraint values of constraint *g*. Therefore, when tests contain non-statistical constraints, *e*_*i*_ in Equation 9 was instead replaced by ei* in Equation 10.

### Test Assembly Procedure

After all experimental conditions are set up, the program of test assembly, written under the NWADH (see Equations 6–15), was run to assemble test. We briefly describe the assembly procedure step-by-step as follows:

First: Take the hard pathway as an example; the test assembly program is based on the initial items in the first stage to find the new item in item bank. The new item needs to have the largest ei* value in the remaining item bank, and ei* was calculated by Equation 10.

Second: When the item with the largest ei* was selected to the hard pathway, we will select the next new item based on the new item and initial item of the first stage. The next new item also needs to have the largest ei* in the remaining item bank.

Third: Repeat the above two steps until the test length meets the experimental requirements. It should be noted that each item was selected only once, which means that the selected new item needs to be removed from the remaining item bank.

## The General Cognitive Diagnosis Models: The G-DINA Model

Cognitive diagnosis models play an important role in CD. They connect examinees’ external response and internal knowledge structure. We need to select the appropriate cognitive diagnostic models for the test to ensure the accuracy and effectiveness of the test.

Generalized DINA (G-DINA; de la Torre, 2011) is an expansion of the DINA model (Deterministic-in-put, noisy-and-gate model; Haertel, 1984; Junker and Sijtsma, 2001). It considers that examinees with different attribute mastery patterns have different probability attributes. For G-DINA, $K_{j}^{*}=\sum_{k=1}^{K}q_{jk}$, where $K_{j}^{*}$ is the number of attributes *k* of item *j*. The G-DINA model divides examinees into $2^{k_{j}^{*}}$ categories and let $a_{lj}^{*}$ denote the reduced attribute mastery patterns based on the measurement attributes of item *j*, $l=1,2,\ldots ,2^{k_{j}^{*}}$. The G-DINA model has different mathematical expressions depending on the function. The three main link functions are the identify link function, logit link function, and log link function. de la Torre (2011) pointed out that the G-DINA model based on the identify link function is a more general form of the DINA model, and its mathematical equation is:

(16) $$P\left(X_{ij}=1|\alpha_{lj}^{*}\right)=\delta_{j0}+\sum_{k=1}^{K_{j}^{*}}\delta_{jk}\alpha_{lk}+\sum_{k^{\prime }=k+1}^{K_{j}^{*}}\sum_{k=1}^{K_{j}^{*}-1}\delta_{jkk^{\prime }}\alpha_{lk^{\prime }}+\ldots +\delta_{j12\ldots k^{*}}\prod_{k=1}^{K_{j}^{*}}\alpha_{lk}.$$

δ_*j*0_ denotes the intercept of item *j*. That is, if examinees do not master all the attributes measured by an item, the value is a non-negative value. δ_*jk*_ is the main effect for α_*k*_. δ_*jkk*′_ is the interaction effect between α_*k*_ and $\alpha_{k^{\prime }}$. $\delta_{j12\ldots k_{j}^{*}}$ denotes the interaction effect from α_1_,…,$\alpha_{k_{j}^{*}}$.

## Simulation Study

### Simulation Design

#### Generated Item Bank

In the simulation study, the number of attributes and the test length were set to five attributes and 21/25 items, respectively. The number of panels were fixed to five or 10 panels. Therefore, there were 2 (the test length) × 2 (the number of panels) = 4 total conditions for this study. Across the conditions, we generated an item bank with 1,000 items. For both IRT and CDM, the measurement of reliability requires a certain test length to ensure that the test reliability can be accurately measured. The test length in the study is based on the CAT and MST. In general, 21 items can provide a good test information in CAT. At the same time, the test is usually divided into three or four stages in MST, and each stage with five or seven items. Therefore, the test length was set to 21 and 25 items in the study.

#### Divided the Item Difficult

For the item difficult level of divide, we referred to the approach of MST. In MST, the item difficult level is divided by the theta parameters because the item difficult parameters and the theta parameters are in the same scale in IRT framework. More specifically, the method is averaged to divide the theta value from large to small into three intervals, and three different intervals represent three different difficulty pathways: easy, medium, and hard pathways. So, we used the same method to divide the difficulty in CD-MST. In the study, item difficulty called *Diff*_*j*_ was described as the mean correct response probability of all KSs of one item. The *Diff*_*j*_ is a probability between 0 and 1. According to the value of *Diff*_*j*_ from item bank, three cut-points were averaged and generated from max *Diff*_*j*_ 0.74 to min *Diff*_*j*_ 0.24 (see Equation 1). We can classify items into easy (0.58–0.74), medium (0.42–0.57), and difficult (0.24–0.41) intervals for CD-MST. The difficult interval with a low value represents the hardest item set. The easy interval with a large value represents the easiest item set.

#### Set Reliability Criteria

Templin’s attribute reliability index is a probability between 0 and 1. Educational Testing Service (2018) proposed 0.9 as representing a very good reliability in CDM. In order to guarantee the test reliability, we chose a high value of 0.9 as the reliability criteria. Therefore, the attribute reliability higher than 0.90 was set as the target reliability value for each attribute.

#### Set the First Stage

In the study, each panel contained three stages. The number of items in each stage is listed in Table 1. It is worth noting that items in the first stage only measured one attribute, whose purposes are to prove the parameters identifiability of CD models (Xu and Zhang, 2016) in the early stage and to improve the classification accuracy of attributes.

**TABLE 1:** Number of items in each stage.

	21 items for test length			25 items for test length		
Pathway	Stage 1	Stage 2	Stage 3	Stage 1	Stage 2	Stage 3
Easy	5	8	8	5	10	10
Medium	5	8	8	5	10	10
Hard	5	8	8	5	10	10

#### Set Quantitative Targets

Quantitative targets are defined as the target attribute reliability proposed by Templin and Bradshaw (2013). The target attribute reliability of each attribute was set to 0.90. The non-statistical constraints in each panel are listed in Table 2, and it should be noted that the test assembly needed to meet the minimum limit constraints. For example, the content balance was divided into four categories, where each category contained at least four items after the test was completed.

**TABLE 2:** Number of non-statistical constraints in test assembly.

Constraints group	Categories	Constraints
Content balance	4	4
Item types	2	8
Answer balance	4	4
Enemy items	1	0
The number of each attribute	5	3

#### Set Assembly Strategy

The top–down strategy was used to assemble the panels, so the non-statistical constraints and quantitative targets would remain parallel between the pathways. R (Version 3.5.1 64-bit; R Core Team, 2018) was used to write the test assembly program under the NWADH.

### Simulation Process

Step 1: Knowledge states. In the study, the test included five independent attributes, and all possible KSs were 2^5^ = 32. The KS of 1,000 examinees was randomly generated from 32 KSs.

Step 2: Q-matrix. The item bank included 1,000 items, and the Q-matrix was randomly generated from 25 to 1 = 31 item attribute patterns.

Step 3: Item parameters. It was generated by the GDINA package (Version 2.1.15; Ma and de la Torre, 2017) in R (Version 3.5.1 64-bit; R Core Team, 2018). According to de la Torre (2011), the item parameters of the G-DINA model are simulated according to *P_j_*(0) and 1-*P*_*j*_(1), and *P_j_*(0) represents the probability of examinees who do not master any attribute required by item *j* and correctly respond to item *j*, 1-*P*_*j*_(1) represents the probability of examinees who master all the attributes required by item j with wrong response to item *j*. Here, the parameters *P*_*j*_(0) and 1–*P*_*j*_(1) were randomly generated between uniform (0, 0.25). This simulation study was replicated 100 times.

Step 4: Test assembly. After all experimental conditions are set up, the program of test assembly, written under the NWADH (see Equations 6–15), was run to assemble the test.

### Evaluation Criteria

For this simulation study, some criteria were computed to evaluate the target attribute reliability violated and the number of constraints violated on each test pathway. The index of the target attribute reliability violated is expressed as:

(17) $$D_{ik}=R_{ik}-T_{ik},$$

where *R*_*ik*_ is the observed reliability of attribute *k* on pathway *i*, *T*_*ik*_ is the target reliability of attribute *k* on pathway *i*, and *D*_*ik*_ represents the difference between the observed reliability and the target reliability.

The number of constraints violated on each constraint is computed as:

(18) $$V=\sum_{i=1}^{N}V_{i},$$

where *V_i_* represents the number of constraints violated, *N* is the constraint number of each test pathway, and *V* is the constraint number for the test pathway.

Other criteria were reported in the results, for example, the item difficulties based on CD, the item difficulties based on the Rasch model, the expected number-correct score based on CD, and the Cronbach *α* coefficient based on the CTT on each test pathway.

### Results

**FIGURE 2:**
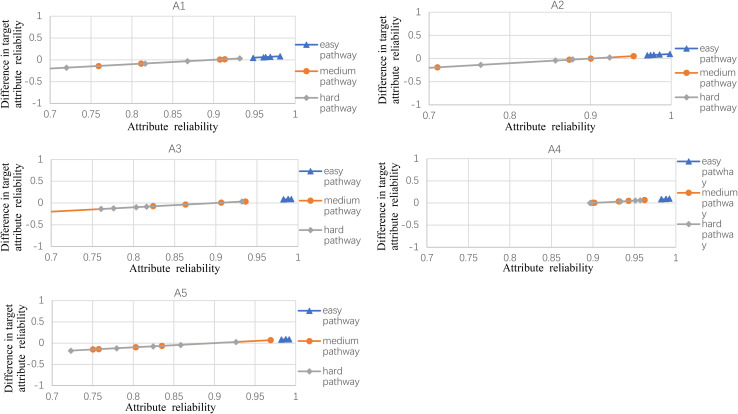
The difference between observed and target reliability with five-attribute, 21-item, and five-panel conditions.

**FIGURE 3:**
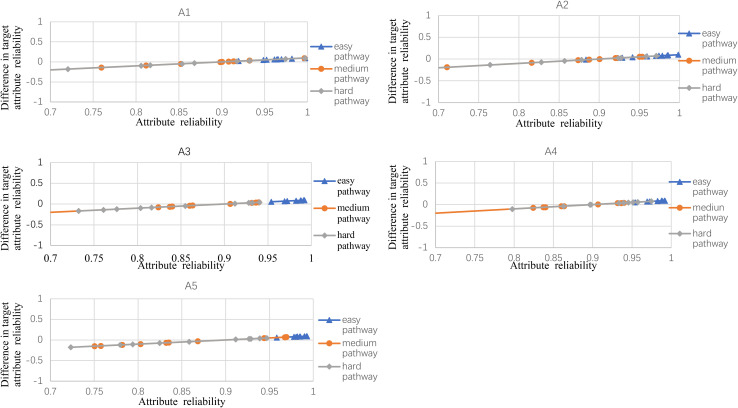
The difference between observed and target reliability with five-attribute, 21-item, and 10-panel conditions.

**FIGURE 4:**
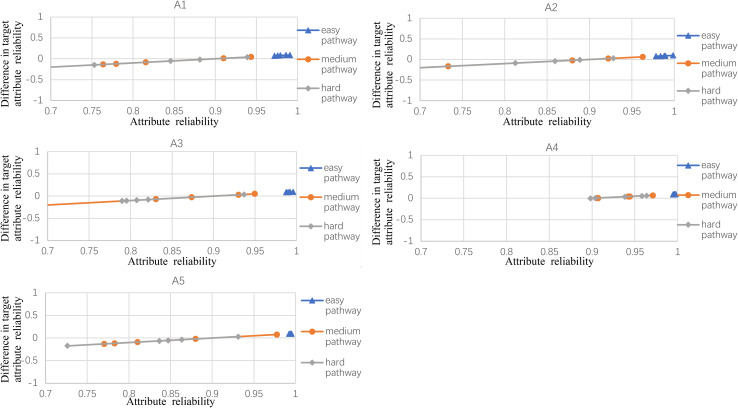
The difference between observed and target reliability with five-attribute, 25-item, and five-panel conditions.

**FIGURE 5:**
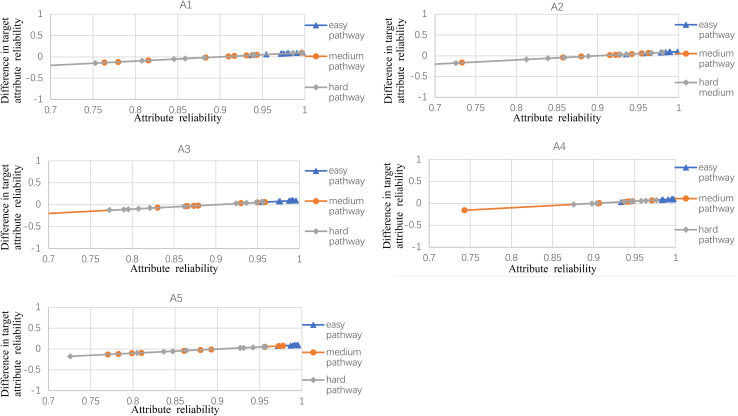
The difference between observed and target reliability with five-attribute, 25-item, and 10-panel conditions.

Figures 2–5 documented the results of the difference between the observed and the target attribute reliability (i.e., *D*_*ik*_; see Equation 17) under four experimental conditions. In Figures 2–5, the points *D*_*ik*_ represent the difference values between the target attribute reliability and the experimental reliability value, and the lower *D*_*ik*_ value indicates a smaller test error. It means that the observed reliability is closer to the target reliability 0.9. Three lines represent different difficulty pathways. We also presented the difference value under different experimental conditions in Figures 2–5.

Figure 2 shows the experimental condition results for five attributes, 21 items, and five panels, and A1–A5 represent attributes 1–5, respectively. Each attribute reliability in each main pathway reached about 0.9, and all the differences between the observed and target reliability were within +0.2. It indicated that the quantitative targets were satisfied. The results of the three other experimental conditions (see Figures 3–5) were very similar to the above experimental condition. Besides, the attribute reliabilities (see Figures 2–5) had slight differences under different item lengths. More detailed, the attribute reliability with 25 items was slightly higher than 21 items, which indicated that the item length affected the attribute reliability, and this result verifies that the test length also affects reliability in CD-MST.

Table 3 summarizes the item statistics for the three primary pathways in different experimental conditions. First, we show the item difficulty of different pathways. The results indicated that item difficulty, in the same simulation data, was very different among three primary pathways in the CDM and the IRT Rasch model. More specifically, the hard pathway with more difficult items has lower *Diff*_*j*_ values (mean correct response probability of all KSs) than those of medium and easy pathways. The medium pathway had a lower value of *Diff*_*j*_ than that of the easy pathway. It should be noted that the lower *Diff*_*j*_ values represent the harder item difficulty in this study. Moreover, the three pathways also show the difference of item difficulty in IRT Rasch model. For example, the item difficulty in the hard pathway is higher than those of the medium and easy pathways. Therefore, these results show that the proposed *Diff*_*j*_ can describe the item difficulty of CDM and can be verified by IRT.

**TABLE 3:** Item difficulties and expected number-correct score statistics for each pathway.

					Expected number-								Expected number-		
Five panels	Cognitive diagnosis		Rasch		correct score		Cronbach α	10 panels	Cognitive diagnosis		Rasch		correct score		Cronbach α
25 items	item difficulties		item difficulties		Based on CD		coefficient	25 items	item difficulties		item difficulties		Based on CD		coefficient
Pathway	Mean	SD	Mean	SD	Mean	SD		Pathway	Mean	SD	Mean	SD	Mean	SD	
Easy	0.6171	0.0125	–0.3868	0.0930	77.092	43.976	0.960	Easy	0.6148	0.0097	−0.9041	0.1329	153.493	83.948	0.978
Medium	0.4593	0.0143	0.5428	0.0895	57.587	28.532	0.924	Medium	0.4772	0.0213	0.2075	0.1261	119.492	59.728	0.962
Hard	0.3794	0.0150	1.0456	0.0924	47.561	27.548	0.916	Hard	0.3887	0.0143	0.9899	0.1342	97.276	56.009	0.958

					**Expected number-**								**Expected number-**		
**Five panels**	**Cognitive diagnosis**		**Rasch**		**correct score**		**Cronbach α**	**10 panels**	**Cognitive diagnosis**		**Rasch**		**correct score**		**Cronbach α**
**21 items**	**item difficulties**		**item difficulties**		**Based on CD**		**coefficient**	**21 items**	**item difficulties**		**item difficulties**		**Based on CD**		**coefficient**
**Pathway**	**Mean**	**SD**	**Mean**	**SD**	**Mean**	**SD**		**Pathway**	**Mean**	**SD**	**Mean**	**SD**	**Mean**	**SD**	

Easy	0.6120	0.0139	–0.4374	0.0956	64.162	37.182	0.952	Easy	0.6099	0.0101	–0.9456	0.0849	127.912	71.358	0.974
Medium	0.4605	0.0138	0.5019	0.0950	48.533	24.348	0.910	Medium	0.4788	0.0214	–0.2315	0.0823	100.812	51.264	0.955
Hard	0.3845	0.0139	1.0091	0.0979	40.411	22.988	0.900	Hard	0.3932	0.0133	0.3654	0.0849	82.583	48.242	0.950

In addition, the standard deviation (SD) of *Diff*_*j*_ in each primary pathway was very small for all experimental conditions, which showed that the items in the same pathway had very similar difficulty levels. We also used the same data to verify the IRT difficulty via Rasch model, which results indicated that the two types of difficulty parameters (IRT and CD) were very consistent. From the above results, it is reasonable to use the mean correct probability of all KSs as the item difficulty index for CD-MST.

Table 3 also displayed that the mean expected number-correct scores were calculated under a large sample with 1,000 examinees. It was shown in the sixtth and seventh columns of Table 3. First, we calculated each examinee’s expected number-correct score in each primary pathway. Then we calculated the mean and SD. As expected, examinees had the highest mean expected number-correct scores in the easy pathway, while they had the lowest mean expected number-correct scores in the hard pathway. It is theoretically reasonable because examinees usually get more scores on easy items.

In Table 3, the Cronbach’s α coefficient was used to verify test reliability. The α coefficients varied from 0.900 to 0.978 with an average of 0.945, which indicates that the proposed CD-MST had high reliability. This shows that the assembled test in the study not only satisfies the reliability of CDM but also the reliability of Cronbach’s α coefficient.

Table 4 documents the number of constraints violated in each constraint group, and the constraints rae set in Table 2. As known in Table 2, the constraint group involved 16 categories and 64 constraints. Table 4 shows that only three of 64 constraints were not satisfied. Specifically, one content balance was not satisfied in the medium pathway with the condition of 21 items and 10 panels, and two answer balances were not satisfied in the hard pathway of the condition of 21 items and 25 items with 10 panels. The overall non-statistical constraint violation rate was about 4.7%, which was an acceptable range. The results indicated that the proposed test assembly had a very good performance in the non-statistical constraints for CD-MST.

**TABLE 4:** Number of constraints violated in each constraint group for each test pathway.

10 panels, 21 items				10 panels, 25 items				5 panels, 21 items				5 panels, 25 items			
Constraint	Easy	Medium	Hard	Constraint	Easy	Medium	Hard	Constraint	Easy	Medium	Hard	Constraint	Easy	Medium	Hard
group	pathway	pathway	pathway	group	pathway	pathway	pathway	group	pathway	pathway	pathway	group	pathway	pathway	pathway
Content category	0	1	0	Content category	0	0	0	Content category	0	0	0	Content category	0	0	0
Item types	0	0	0	Item types	0	0	0	Item types	0	0	0	Item types	0	0	0
Answer keys	0	0	1	Answer keys	0	0	1	Answer keys	0	0	0	Answer keys	0	0	0
Attribute times	0	0	0	Attribute times	0	0	0	Attribute times	0	0	0	Attribute times	0	0	0
Enemy item	0	0	0	Enemy item	0	0	0	Enemy item	0	0	0	Enemy item	0	0	0

## Conclusion and Discussion

The MST with the advantages of P&P and CAT is to be applied to many large-scale international examinations. However, the existing MST with the IRT focuses on the examinees’ general ability and cannot provide further detailed diagnostic information. Because CD mainly focuses on the multidimensional and discrete cognitive attributes, some test assembly indexes in MST (such as the item difficulty and the Fisher information) are not suitable for CD-MST. There has been no recent research on CD-MST. Although some studies (such as Zheng and Chang, 2015) provided on-the-fly MST (OMST; Zheng and Chang, 2015), which may be a practical method of CD-MST, this may lead to many problems, such as (1) the test developer having difficulty in managing tests before administering, (2) the parallel of the test is difficult to ensure, (3) and the non-statistical constraint also is difficult to satisfy. To address the above issues, a CD-MST framework that not only provides rich diagnostic information about the candidates but also retains the inherent advantages of MST was proposed in this paper. This paper also proposed and employed two statistical indexes, namely, item difficulty and attribute reliability, as the statistical constraints of CD-MST. In this paper, the proposed item difficulty index is a good indicator of the item difficulty based on CD, which has a very significant high correlation with the item difficulty parameter based on IRT (such as the Rasch model). The reliability index also guarantees the reliability and measurement error of tests. These indexes can provide statistical information, which makes it possible to automate test assembly for CD-MST. At the same time, the results showed that the NWADH algorithm under the CD framework successfully satisfied the non-statistical constraints. It showed that the proposed CD-MST framework and statistical indicators are acceptable for CD-MST.

This study employed the NWADH heuristic method to assemble the CD-MST under ATA. The results showed that the statistical and non-statistical constraints were both well satisfied, and the assembled test panels were parallel overall. At the same time, the non-statistical constraints (such as the attribute balance and content balance) were fully considered in CD-MST, which helps improve the content validity and structural validity of CD-MST. Therefore, the proposed CD-MST with NWADH heuristic algorithms not only provides rich diagnostic information but also retains the advantages of MST.

## Limitations and Further Research

As an early exploration of CD-MST, despite the promising results, there are still some limitations that need to be studied further. First, even though the CD item difficulty index, the mean correct probability of all KSs, fully represents the item difficulty, it is verified by the IRT model. Further research also can develop other indexes to measure the item difficulty in CDM. For example, Zhan et al. (2018) proposed the probabilistic-input, noisy conjunctive (PINC) model, which defined attribute mastery status as probabilities and reported the probability of knowledge status for examinees from 0 to 1. According to Zhan et al. (2018), classifying an examinee’s KSs to 0 or 1 will cause a lot of information of examinees to be lost, so the PINC model can provide more precise and richer information to examinees’ KSs than the traditional CDMs. Therefore, researchers can try to use the probability of examinees’ KSs to develop a new difficulty index in the future.

Second, attribute reliability was regarded as a quantitative target in this study, which is illustrative but not prescriptive. In future studies, other reliability or information/measurement error indicators may also be considered as quantitative targets. For example, the classification accuracy was proposed by Cui et al. (2012), the classification matches were proposed by Madison and Bradshaw (2015), and the classification consistency was proposed by Matthew and Sinharay (2018). In the future, the comparative analysis of these reliability indexes can be applied to the test assembly in CD-MST.

Third, the NWADH method was used in this study to assemble the panels. Although this method can guarantee the successful completion of the test assembly, there is still a small violation of the constraints. For example, content constraints were slightly violated in this study. Even if this violation is allowed in the NWADH method, other methods may be considered to ensure that all constraints are met. In fact, the linear programming method and the Monte Carlo method are also widely used in MST. Although these two methods are influenced by the size and quality of the item bank, they can fully meet the test specification. Besides, Luo and Kim (2018) proposed the mixed integer linear programming (MILP) to assemble tests in MST. The result of the MILP method shows that the method had the advantage of the heuristic algorithm and 0–1 linear programming algorithm. Perhaps, the MILP method is also a reasonable ATA method for CD-MST and can resolute the violence of constraints. Therefore, the development of new methods that can fully meet the constraints and successfully assemble tests is also one of the future research directions.

Finally, the test length also needs to be explored in a further study. In the study, the difference between the reliability and the constraints is not significant. The difference between test length levels can be larger (e.g., 21 vs. 42) and be further studied to explore the impact of test length. Researchers can design the different item numbers to explore the best test length that can provide the maximum information and meet the test constraints.

## Data Availability Statement

The datasets generated for this study are available on request to the corresponding author.

## Author Contributions

GL conceptualized the study, developed the methodology, performed the mathematical derivation, conducted the formal analysis, analyzed the data, wrote the original draft of the study, and wrote the article. GL, DT, and YC edited and reviewed the manuscript. GL, XG, and DW performed the simulation studies. DT acquired the funding and resources, performed the investigation, and supervised the study project. All authors contributed to the article and approved the submitted version.

## Conflict of Interest

The authors declare that the research was conducted in the absence of any commercial or financial relationships that could be construed as a potential conflict of interest.
